# A single center retrospective study on real world CAR T-cell therapy: focus on early hematological toxicity

**DOI:** 10.3389/fmed.2024.1465802

**Published:** 2024-11-18

**Authors:** Vadim Lesan, Konstantinos Christofyllakis, Moritz Bewarder, Lorenz Thurner, Jörg Bittenbring

**Affiliations:** Department of Hematology and Oncology, Saarland University Hospital, Homburg, Germany

**Keywords:** lymphoma, CAR-T, hematological toxicity, Hematotox-Score, early toxicity

## Abstract

Patients with relapsed/refractory diffuse large B-cell lymphoma (DLBCL), mantle cell lymphoma (MCL) and multiple myeloma have poor outcomes. CAR-T completely changed the landscape of therapy options, improving not only response rates but also survival outcomes. Hematological toxicity after chimeric antigen receptor therapy (CAR-T) is of increasing interest, being a recognized prognostic factor in this setting. We report our experience with early hematological toxicity after CAR-T therapy and point some important aspects regarding the Hematotox-Score. We identified a strong negative correlation between Hematotox-Score and platelet count at first day of cytokine release syndrome (CRS). Hematotox-Score was predictive of hemoglobin levels at day 28 after CAR-T. Ferritin remained high after 28 days post CAR-T in patients with high Hematotox-Score. Hematotox-Score did not associate with mortality in our cohort. We did not find any significant association between the hematological parameters (hemoglobin, platelets, and neutrophil counts), ferritin, LDH at first day of CRS and mortality. In conclusion, we demonstrate that Hematotox-Score is predictive of early hematological toxicity after CAR-T. Although, patients with higher degree of hematological toxicities have poorer survival outcomes, Hematotox-Score lacks predictive potential, probably due to its limitations. Further development of hematological scores predicting survival outcome in the context of CAR-T are needed.

## Background

Patients with relapsed/refractory diffuse large B-cell lymphoma (DLBCL), mantle cell lymphoma (MCL) and multiple myeloma have very poor outcomes (1–3). As a consequence, chemotherapy-based therapies are increasingly challenged by cellular therapies. Chimeric antigen receptor therapy (CAR-T) emerged as an important option in this setting, improving not only response rates but also progression free survival and overall survival (4). Although, the success of CAR-T is undoubted, important concerns about the toxicity and non-relapse mortality of CAR-T remain unsolved. Cytokine release syndrome (CRS) and the immune cell effector neurotoxicity syndrome (ICANS) represent the most important early toxicity manifestations after CAR-T (5). CRS rates and grades vary significantly depending on the CAR-T product and disease context. CRS rates can be as high as 80% (6). Grade ≥ 3 CRS rates between 5 and 12% were reported in different real-world studies (6–9). The rates of ICANS after CAR-T are between 33 and 49% (6, 8, 9). Grade 3 or more ICANS rates are substantially lower, ranging from 11 to 14% (6–9). Both CRS and ICANS were found to correlate with non-relapse mortality in CAR-T treated patients (9). One real world study reported that 66% of all patients who died of infection related causes after CAR-T therapy, experienced previously ICANS Grade ≥ 3 (6). Prolonged neutropenia with infection and ICANS, were identified as risk factors for non-relapse mortality after CAR-T (6). Considering these findings, many attempts were taken to reduce the CRS and ICANS rates. The use of early and prophylactic corticosteroids showed decreased rates and severity of CRS and ICANS (10, 11). Recently, a phase II study reported lower CRS and ICANS rates in patient receiving prophylactic anti-IL-1 antibodies (12). With more patients achieving longer survival rates after CAR-T, toxicities like infections and bone marrow suppression become clinically relevant. Recently, EHA and EBMT reported a consensus paper on grading and practice recommendations for immune-effector cell associated hematological toxicity (13). Hematological toxicity is a common adverse effect of CAR-T therapy irrespective of CAR-T product or disease entity (14). CAR-T related hematological toxicity has different patterns of manifestation. As of today, three different patterns were described, with the aplastic one being associated with the worst clinical outcome (15). The Hematotox-Score has been proposed as a tool to identify patients at high risk for prolonged hematological toxicity and can be used in managing hematological toxicity after CAR-T (13). Despite increasing recognition of hematological toxicity and its role in the non-relapse mortality after CAR-T, management these toxicities is difficult. This prompts better risk stratification and prognostic assessment. Here, we take an attempt to define risk factors associated with early hematological toxicities after CAR-T and point some important aspects regarding the Hematotox-Score.

## Materials and methods

### Patients and data collection

In this single center real world study, we included all patients treated with CAR-T products for r/r DLBCL, MCL and multiple myeloma. The treatment period reported here is between April 2022 and February 2024. Toxicity and survival outcomes were assessed in all included patients. Lymphodepleting chemotherapy consisting of fludarabine, and cyclophosphamide was administered according to the CAR-T manufacturers’ recommendations. Clinical data was extracted from medical records of patients after institutional board approval.

### CAR-Hematotox Score

We used the Hematotox-Score calculated prior to lymphodepleting chemotherapy from the German Lymphoma Alliance (GLA) (Supplementary Table 1): https://www.german-lymphoma-alliance.de/Scores.html (16). The calculation, grading, and interpretation of Hematotox-Score was performed according to previous published recommendations (15).

### Toxicity grading and management

CRS and ICANS were graded according to American Society for Transplantation and Cellular Therapy (ASTCT) consensus criteria (Supplementary Tables 2, 3) (17). CAR-T toxicity management was performed according to international and institutional guidelines.

### Statistical analysis

Continuous variables are reported as median and range, while categorical variables as number and percentage. Statistical significance (*P* < 0.05) between groups was determined by nonparametric Mann-Whitney *U* test for continuous variables and Fisher’s exact test for comparison of categorical variables. Regression analysis was performed for multivariate analysis. All statistical analysis was performed with SPSS (IBM SPSS, Chicago, United States of America 2019) and R (The R Foundation for Statistical Computing, Vienna, Austria 2022).

## Results

### Study and patient characteristics

Between April 2022 and February 2024, twenty-seven patients were treated at our tertiary center with CAR-T therapy for r/r DLBCL, mantle cell lymphoma or multiple myeloma.

The median age of all patients was 61 (range: 24–77) years. Sixty percent of patients were male. The median hematopoietic cell transplantation comorbidity index (HCT-CI) of all patients was 2 (range: 0–8). The median Eastern Cooperative Oncology Group Performance Status (ECOG) Score was 1 (range: 0–4). Thirty one percent of patients had an ECOG > 2. Table 1 includes the baseline characteristics of all patients.

**TABLE 1:** Patient baseline characteristics.

Baseline characteristics	Results
Age, median (range)	61 (24–77)
Male, *n* (%)	16 (60)
ECOG, *n* (%)	1 (0–4)
Body mass index, median (range)	25 (17–36)
Diabetes mellitus, *n* (%)	4 (15)
Diffuse large B-cell lymphoma, *n* (%)	20 (74)
Mantel cell lymphoma, *n* (%)	4 (15)
Multiple myeloma, *n* (%)	3(11)
Previous lines of therapies, *n* (%)	2 (1–4)
Autologous stem cell transplantation, *n* (%)	8 (30)
Bulky disease, *n* (%)	10 (37)
Central nervous system disease, *n* (%)	4 (15)
Extranodal disease, *n* (%)	12 (44)

### Baseline characteristics by disease entity

In patients with DLBCL the median number of previous therapy lines was 2 (range: 1–4). The median age was 58.5 (range: 24–77). Fifty five percent of patients were male. The median HCT-CI Score was 2.5 (range: 1–3). Median ECOG was 1 (range: 0–4). Five patients (25%) underwent previous hematopoietic stem cell transplantation. Most patients (74%) had a stadium IV disease at the time of CAR-T therapy. Extranodal disease was present in 63% of patients. Four patients (20%) presented with CNS disease at the time of CAR-T therapy. Thirty five percent of patients had a transformed DLBCL, and forty six percent had a high grade DLBCL. Bulky disease was present in 8 (42%) patients. The median IPI score at the time of CAR-T was 3 (range 0–5). Ninety percent of patients received bridging therapy. Radiotherapy as bridging therapy was performed in only 3 (15%) patients. The median dose of fludarabine was 58.7 mg/m^2^/day (range: 50–65 mg/m^2^/day). The median dose of cyclophosphamide was 970 mg/m^2^/day (range: 840–1090 mg/m^2^/day).

In patients with MCL, the median age was 63.5 (range: 61–65) years. Seventy five percent of patients were male. The median HCT-CI Score was 2 (range 1–3). Median ECOG was 2 (range: 1–4). The median number of therapy lines was 3 (range: 2–4). Seventy five percent of patients received previously an autologous stem cell transplantation. Median stage of the disease was 3 (range: 2–4). Extranodal disease was absent in all patients. Fifty percent of patients presented with high-risk features. Bulky disease was absent in all patients. The median MIPI Score was 7.5 (range 6.5–9). All four patients received a bridging therapy. Radiotherapy was not part of the bridging therapy in any of the patients. The median dose of fludarabine was 58.7 mg/m^2^/day (range: 50–65 mg/m2/day). The median dose of cyclophosphamide was 970 mg/m^2^/day (range: 840–1090 mg/m^2^/day).

The median age of the patients with multiple myeloma was 70 (range: 57–74) years. Sixty six percent of patients were male. All patients had an ECOG of 1. Median HCT-CI Score was 2 (range: 1–8). At the time of CAR-T, all patients had a revised international scoring system (R-ISS) stage III. No patient presented with extramedullary disease. Median number of previous therapy lines was 5 (range: 4–6). All patients received previously autologous stem cell transplantation. At the time of CAR-T, two patients were experiencing progressive disease, and one patient was in complete remission. Bridging therapy was administered in only one patient. The median dose of fludarabine was 51.2 mg/m^2^/day (range: 48.7–60 mg/m^2^/day). The median dose of cyclophosphamide was 510 mg/m^2^/day (range: 490–600 mg/m^2^/day).

### Bridging therapy efficacy

The overall response rate (ORR) after bridging therapy was 47% in our data set. Four (17%) patients achieved a complete remission, seven patients had (30%) a partial remission and eight patients (35%) had progressive disease after bridging therapy.

Twelve patients (54%) fulfilled the eligibility criteria for either ZUMA-1, ZUMA-2, or ZUMA-7 studies (18–20). All three patients with multiple myeloma fulfilled the eligibility criteria for CARTITUDE-1 study (21).

### CAR-T therapy efficacy

At the first disease control assessment around day 28 post CAR-T infusion, 62% of patients were in a complete remission, 10% of patients were in a partial remission and 14% of patients had a progressive disease. Data on remission status is missing or immature in 4 (14%) patients. Six (22%) patients died during the follow up. Four patients died because of disease progression and two patients died because of infection related complications. One patient experienced disease progression beyond day 28 post CAR-T. Eighty percent of the patients who progressed received subsequent therapies. Sixty percent of patient with progressive disease received a CD20/CD3 bispecific antibody and one patient received a second generation Bruton-tyrosine kinase inhibitor.

### Toxicity results

CRS was diagnosed in 93% of patients. Only one (4%) patient had a CRS grade ≥ 3. The median grade of CRS was 1 (range: 0–3). The median time from CAR-T infusion to first CRS manifestation was 1 day (range: 0–6 days). The median time from infusion to maximal grade of CRS was 3 days (range: 0–32 days).

Sixty six percent of patients developed any grade ICANS. Twelve (46%) patients experienced ICANS grade ≥ 3. The median ICANS grade was 2 (range: 0–4). The median time from infusion to first manifestation of ICANS was 4 days (range: 0–19). The median time from infusion to maximal grade of ICANS was 5 days (range: 0–32).

Six patients (22%) received prophylactic corticosteroid therapy before CAR-T infusion. Corticosteroids were administered for a median of 11 days (range: 0–101).

### Hematological toxicity

The rate of all grade anemia at the time of the lymphodepletion was 77%. Both on day 0 of CAR-T and day 28 post CAR-T therapy, the rate of all grade anemia was 88%. The rates of grade ≥ 3 anemia at the time of lymphodepletion, day 0 and day 28 post CAR-T were 7, 7, and 0%, respectively.

The rate of all grade thrombocytopenia at the time of the lymphodepletion was 30%. At the time of CAR-T infusion 51% of patients had thrombocytopenia. At day 28 post CAR-T therapy, the rate of thrombocytopenia was 77%. The rates of grade ≥ 3 thrombocytopenia at the time of lymphodepletion, day 0 and day 28 post CAR-T were 11, 15, and 41%, respectively.

At lymphodepletion, neutropenia was present in 48% of patients. This increased to 96% on CAR-T infusion day. At day 28 post CAR-T therapy, the rate of neutropenia was 70%. The rates of grade ≥ 3 neutropenia at the time of lymphodepletion, day 0 and day 28 post CAR-T were 15, 66, and 42%, respectively.

**FIGURE 1:**
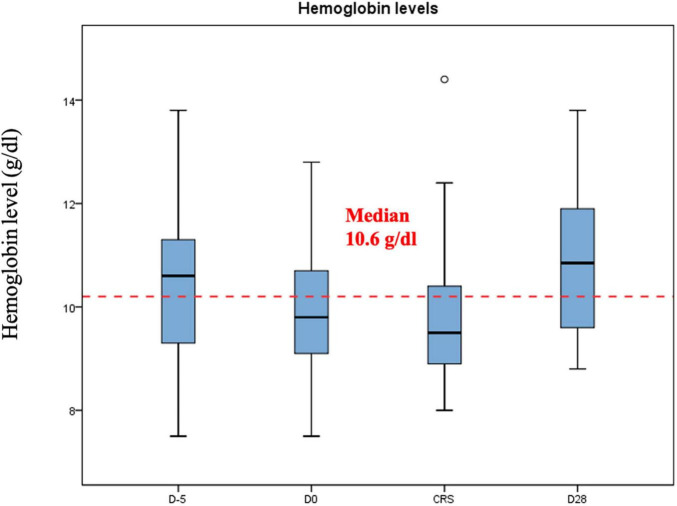
Hemoglobin levels (g/dl) during CAR-T.

**FIGURE 2:**
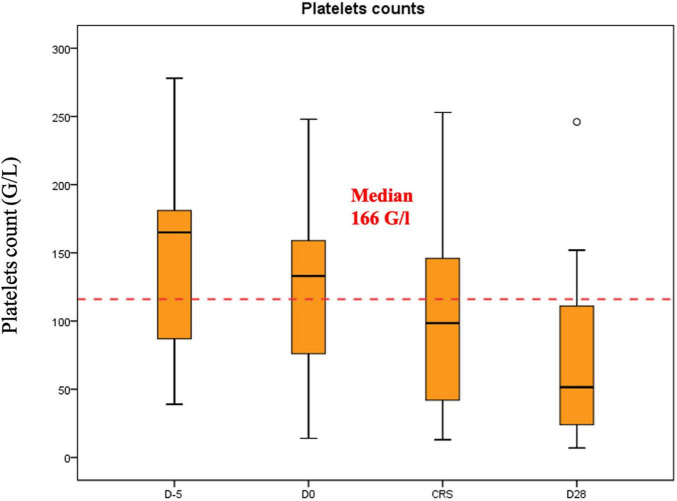
Platelet counts (G/L) during CAR-T.

**FIGURE 3:**
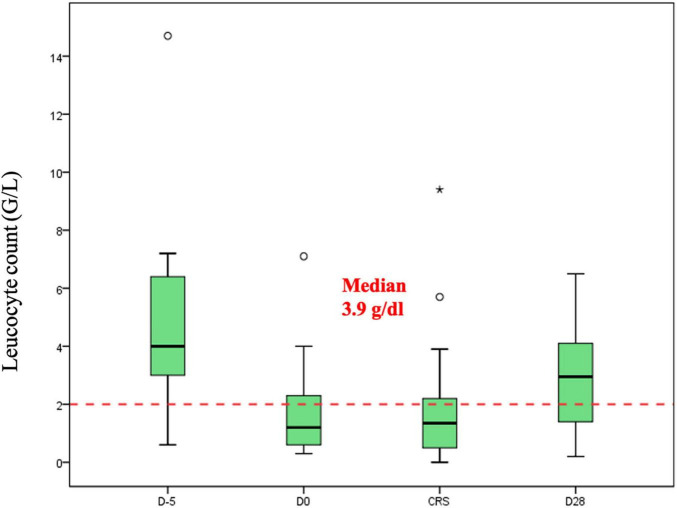
Leucocyte counts (G/L) during CAR-T.

Median hemoglobin, platelet and leucocyte counts at the beginning of the lymphodepletion were 10.6 g/dl (range: 7.5–13.8 g/dl), 166 G/l (range: 39–278 G/l) and 3.9 G/l (range: 0.6–14–7 G/l), respectively. In Figures 1–3 we represent the box plots of the hemoglobin level, platelet and leukocyte counts over the study period.

Markers of disease activity including LDH, and ferritin were monitored alongside hematological parameters. As such, median LDH at lymphodepletion was 199 U/I (range: 124–906 U/I). Median ferritin at the day of CAR-T infusion was 1035 mg/dl (range: 19–13.337 mg/dl). Median reactive protein c (CRP) at CAR-T infusion day was 11.5 mg/dl (range: 2–246 mg/dl).

### Hematotox-Score and hematological toxicity

Median Hematotox Score was 2 (0–6). Ten (37%) patients had a Hematotox-Score ≥ 2. Four (18%) patients with prolonged hematological toxicity received a stem cell boost to improve the bone marrow function. Hypogammaglobulinemia was present in 14 (64%) patients.

In an exploratory analysis we looked at the correlation between Hematotox-Score, hematological toxicity and inflammation at the first day of CRS. We chose this timepoint as it represents the time were immunosuppressive therapies like corticosteroids and/or anti-IL-6 antibodies are usually started. We identified a strong negative correlation between Hematotox-Score and platelet count at first day of CRS (*r* = −0.71, *p* = 0.001). This correlation was attributed mainly to low platelet counts before lymphodepletion, which explained most of the observed correlation. Neutrophil counts at first day of CRS showed a negative correlation with the Hematotox-Score (*r* = −0.58, *p* = 0.001). Ferritin correlated positively with Hematotox-Score (*r* = 0.6, *p* = 0.001).

**FIGURE 4:**
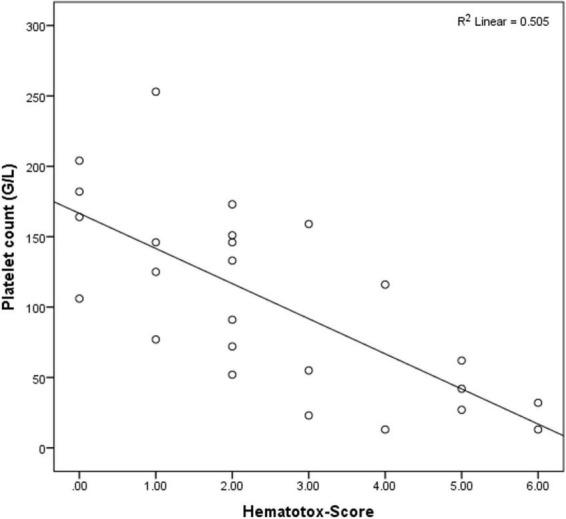
Correlation between Hematotox-Score and platelet counts at first day of CRS.

Figure 4 shows the strong correlation between Hematotox-Score and platelet counts at first day of CRS. Fludarabine and cyclophosphamide doses did not show any correlation with the hematological parameters at any time point during CAR-T therapy.

Hematotox-Score was predictive of hemoglobin levels at day 28 after CAR-T. Patients with higher Hematotox-Score had lower hemoglobin levels at day 28 after CAR-T (*r* = −0.5, *p* = 0.01). Ferritin was higher at day 28 after CAR-T in patients with higher Hematotox-Score (*r* = 0.6, *p* = 0.003).

In the univariate analysis, mortality after CAR-T associated significantly with lower platelet counts at day 28 post CAR-T (*p* = 0.02), higher LDH (*p* = 0.04) and ferritin (*p* = 0.005) at first day of CRS, higher rates of CRS Grade ≥ 3 (*p* = 0.04), prolonged treatment with corticosteroids (*p* = 0.005), and the use of stem cell boost (*p* < 0.01). Hypogammaglobulinemia was another factor significantly associated with CAR-T mortality (*p* = 0.006).

In a multivariate logistical analysis, we looked at the hematological factors associated with mortality after CAR-T. Hematotox-Score did not associate with mortality in our cohort. We did not find any significant association between the hematological parameters (hemoglobin, platelets, and neutrophil counts), ferritin, LDH at first day of CRS, and mortality.

## Discussion

### Key findings

In this retrospective study we observed high rates of early hematological toxicity after CAR-T therapy in a real-world monocentric patient cohort. We demonstrated that patients with higher Hematotox-Score have the lowest platelet and neutrophil counts at the first day of CRS, putting this cohort of patients at a very high risk of complications. Furthermore, we demonstrate that Hematotox-Score is predictive of the hemoglobin and ferritin level up to day 28 after CAR-T. Finally, we found that Hematotox-Score does not correlate with mortality rate in our study. Altogether, our results confirm previous reports on hematological toxicity in patients treated with CAR-T (15). Some important lessons derive from our observations.

Firstly, baseline thrombocytopenia strongly correlated with thrombocytopenia at day 28 after CAR-T. This probably reflects an impaired hematological reserve, due to direct cytotoxic effect of previous lines of chemotherapies. Interestingly, we found that 75% of patients who needed a stem cell boost for poor hematological function, had low platelets counts before lymphodepletion. All these patients previously received hematopoietic stem cell transplantation and had a median of three lines of therapy.

Secondly, Hematotox-Score correlates with ferritin levels at day 28 post CAR-T. This fact supports the direct inflammatory damage to the stem cell niche hypothesis, which is reflected in higher ferritin levels. The inflammation caused by the cytokine release interferes with the normal bone marrow function through high levels of circulating cytokines.

Thirdly, although Hematotox-Score can predict hematological toxicity, it does not predict mortality in our study. This means, that other important factors like disease status, response to CAR-T and comorbidities determine the outcome of these patients.

Hematotox-Score was developed to comprehend two theories of hematological toxicity in patients undergoing CAR-T: the depleted bone marrow theory (either due to previous chemotherapies or due to bone marrow involvement of the disease) and the hyperinflammation theory (13, 15). Indeed, our cohort of patients presented a high Hematotox-Score before lymphodepletion, both due to decreased bone marrow reserves and increased inflammation. In our study almost all patients developed CRS. This fact precluded a direct comparison between patients with CRS and without CRS regarding the impact of bone marrow reserve and hyperinflammation on hematological toxicity.

Although, the Hematotox-Score is an important tool in assessing patients at risk for hematological toxicities, in our view, it has some important limitations. Firstly, it is a static measure performed at only one time point during a complicated and continuously changing therapy strategy. This might omit important factors after lymphodepletion, which may influence the hematological outcome of the patients. Secondly, the cut off value for each variable included in the score are chosen to maximize the predictive value regarding the hematological toxicity and not important outcomes like overall survival. This fact explains why Hematotox-Score does not correlate with survival outcome. Further studies are needed to assess the meaningfulness of these cut off values in the CAR-T setting. Thirdly, all variables in the score are indirect measure of either bone marrow function or inflammation and might not represent the true biological effects in this context.

### Limitations

Our study has some limitations. The retrospective nature of the study could have introduced some biases. The monocentric patient cohort reflects our local management, diagnosis and therapy of CAR-T toxicities strategies and does not necessary reflect international standards., The number of the patients treated with CAR-T is relatively low. This could have masked and/or inflated some of the presented results.

## Conclusion

In conclusion, we demonstrated that Hematotox-Score is predictive of early hematological toxicity after CAR-T. Although, patients with higher degree of hematological toxicities have poorer survival outcomes, Hematotox-Score lacks predictive potential for survival, probably due to its limitations. Further development of hematological scores predicting survival outcome in the context of CAR-T are urgently needed.

## Data Availability

The raw data supporting the conclusions of this article will be made available by the authors, without undue reservation.
