# Supplementary material for: A single center retrospective study on real world CAR T-cell therapy: focus on early hematological toxicity
Source: Front Med (Lausanne). 2024 Nov 18;11:1465802. doi: 10.3389/fmed.2024.1465802 (PMC11608982; doi:10.3389/fmed.2024.1465802)
Supplement: Supplementary file 1 [file Table_1.DOCX]

| **Baseline characteristic** | **0 Point** | **1 Point** | **2 Points** |
| --- | --- | --- | --- |
| **Platelet count** | >175.000/ μL | 75.000-175.000/ μL | <75.000/ μL |
| **Absolute neutrophil count** | >1200/ μL | <1200/ μL |  |
| **Hemoglobin** | >9 g/dl | <9 g/dl | - |
| **C-reactive Protein** | <3 mg/dl | >3 mg/dl | - |
| **Ferritin** | <650 ng/ml | 650-2000 ng/ml | >2000 ng/ml |

**Table.1 Hematotox-Score**

**Table.2 CRS Grading according to American Society for Transplantation and Cellular Therapy**

| **CRS Parameter** | **Grade 1** | **Grade 2** | **Grade 3** | **Grade 4** |
| --- | --- | --- | --- | --- |
| **Fever** | Temperature 38°C | Temperature 38°C | Temperature 38°C | Temperature 38°C |
|  |  |  |  |  |
| **Hypotension** | None | Not requiring vasipressors | Requiring vasopressors with or without vasopressin | Requiring multiple vasopressors ( excluding vasopressin) |
|  |  |  |  |  |
| **Hypoxia** | none | Requiring low-flow nasal canule or blow by | Requiring high flow  nasal cannula or facemask, nonrebreather mask or Venturi mask | Requiring positive pressure (eg. CPAP, BIPAP, intubation, mechanical ventilation) |

**Table.3 ICANS Grading according to American Society for Transplantation and Cellular Therapy**

| **Neurotoxicity Domain** | **Grade 1** | **Grade 2** | **Grade 3** | **Grade 4** |
| --- | --- | --- | --- | --- |
| **ICE Score** | 7-9 | 3-6 | 0-2 | 0 |
| **Depressed level of consciousness** | Awakens spontaneously | Awakens to voice | Awakens only to tactile stimulus | Patient is unarousable or requires vigorous or repetitive  stimuli to arouse. Stupor or coma |
| **Seizure** | N/A | N/A | Any clinical seizure focal or gen- eralized that resolves rapidly or  nonconvulsive seizures on EEG that resolve with intervention | Life-threatening prolonged seizure (>5 min); or Repetitive clinical or electrical seizures without return to baseline in between |
| **Motor findings** | N/A | N/A | N/A | Deep focal motor weakness such as hemiparesis or paraparesis |
| **Elevated intracranial pressure/ brain edema** | N/A | N/A | Focal/local edema on neuroimaging | Diffuse cerebral edema on neuroimaging; decerebrate or decorticate posturing; or cranial nerve VI palsy; or papilledema; or Cushing's triad |
